# Word Frequency Is Associated With Cognitive Effort During Verbal Working Memory: A Functional Near Infrared Spectroscopy (fNIRS) Study

**DOI:** 10.3389/fnhum.2019.00433

**Published:** 2019-12-13

**Authors:** Amy Berglund-Barraza, Fenghua Tian, Chandramalika Basak, Julia L. Evans

**Affiliations:** ^1^School of Behavioral and Brain Sciences, The University of Texas at Dallas, Dallas, TX, United States; ^2^Department of Bioengineering, The University of Texas at Arlington, Arlington, TX, United States

**Keywords:** working memory, word frequency, functional near-infrared spectroscopy, cognitive effort *n*-back, prefrontal cortex

## Abstract

**Purpose:**

Psycholinguistic models traditionally view verbal working memory capacity as independent from linguistic features; connectionist models suggest otherwise. Moreover, lexical processing studies show high frequency words differ in cognitive effort from low frequency words, although these effects during concurrent processing of words in working memory are unknown. This novel study examines potential differences in cognitive effort, as measured by differences in HbO2 and Hb, for high frequency versus low frequency words during a working memory paradigm.

**Methods:**

A total of 21 neurologically typical participants (age 18–23) completed an auditory, *n*-back, working memory task comparing performance with high- as compared to low- frequency words. Hemodynamic changes in the prefrontal cortex were recorded with a continuous-wave functional near-infrared spectroscopy (fNIRS) device. Behavioral data (accuracy, reaction time) were recorded using E-prime.

**Results:**

Differences in word frequency were evident at both behavioral and neurological levels. Participants were more accurate, albeit slower in identifying the target two back in a sequence for low- as compared to high-frequency words. Patterns of hemodynamic changes were also significantly different between HF and LF conditions.

**Conclusion:**

The results from this study indicate that the behavioral and neurological signatures inherent in holding high- versus low-frequency words in working memory differs significantly. Specifically, the findings from this study indicated that words differing in frequency place different demands on cognitive processing load in memory updating tasks.

## Introduction

Working memory, the ability to store and manipulate information in the mind for short periods of time, supports various cognitive functions, including the acquisition and further use of language (Baddeley, 2003; Szmalec et al., 2012; Squire and Dede, 2015). Verbal working memory, the portion of working memory that stores and manipulates auditory linguistic information, has been directly linked to sentence comprehension in both typical and atypical individuals. Namely, higher verbal working memory ability correlates with higher sentence comprehension abilities, and in the case of children, later linguistic ability (Caplan and Waters, 1999; Montgomery, 2000). The importance of working memory for language spurred investigations into what types of linguistic factors impact verbal working memory abilities. The focus of verbal working memory research has been on the speed and accuracy with which a listener can retrieve stored information from memory after some brief interval. However, it could be argued that this research has focused primarily on the short-term memory portion of verbal working memory with the assumption that results pertaining to short-term memory would generalize to the larger working memory system. While short-term memory has traditionally been thought of as a storage device permitting the listener to simply hold items until they are to be recalled, with emphasis placed on the storage and retrieval of information after a brief interval, working memory has been viewed as both storage of items for later retrieval as well as the storage of partial results necessary for complex sequential computations such as language comprehension. Although some models consider short-term and working memory as distinct forms of memory, most view short-term memory and working memory interchangeably as the simultaneous storage and manipulation of information (Baddeley, Cowan, Carpenter, Just etc.). We take the view that short-term and working memory are integrally linked, with short-term memory being the temporary storage component of the larger working memory system.

Working memory models differ in the extent to which memory and extant language knowledge are treated as distinct constructs. Some models argue for a division of labor between long-term storage of lexical representations and the management of verbal information in real-time (Baddeley, 2002, 2010), while others argue for an embedded framework where “working memory” is not a separate construct but the temporary activation of long-term memory under the focus of attentional control (i.e., Cowan, 1999, 2008). Specifically, the embedded models assume that verbal working memory capacity cannot be separated from knowledge representations and that individual differences in verbal working memory capacity are due, not to differences in capacity, but to differences in activation levels of lexical representations in the listener’s lexicon which manifest as differences in behavioral speed and accuracy of lexical processing, and retrieval and subsequent accuracy and efficiency of sentence comprehension (Just and Carpenter, 1992).

For example, in spoken word recognition studies, word frequency effects can be seen in listeners’ quicker reaction times and greater accuracy in accessing high-frequency as compared to low-frequency words from their lexicon, in recalling high- as compared to low-frequency word in serial recall tasks, and recognizing high- as compared to low-frequency words in lexical decision tasks (e.g., Barry et al., 1997; Hulme et al., 1997; Gerhand and Barry, 1998, 1999; Luce and Pisoni, 1998; Roodenrys et al., 2002; Mainela-Arnold and Evans, 2005; Mainela-Arnold et al., 2010a). Word frequency also influences spoken word recognition, with listeners requiring significantly less acoustic information to recognize high-frequency as compared to low-frequency words in the speech stream (Grosjean, 1980; Walley et al., 1995; Metsala, 1997; Mainela-Arnold and Evans, 2005; Mainela-Arnold et al., 2010b).

It has been argued that differences in the strength of long-term associative links inherent in word frequency at the whole word level is the reason why word frequency influences the speed and accuracy of lexical processing (Stuart and Hulme, 2000; Saint-Aubin and LeBlanc, 2005). Specifically, the higher the frequency of the whole word, the stronger the long-term associative links that translates into higher resting activation levels, which in turn then translates into greater ease of access from the lexicon (McClelland and Elman, 1986; Stuart and Hulme, 2000; Saint-Aubin and LeBlanc, 2005). There is indirect evidence from neuroimaging studies that high-frequency words have higher resting state activation levels and require less computational resources to access from memory as compared to low-frequency words (cf. Polich and Donchin, 1988; Chee et al., 2002; Fiebach et al., 2002). For instance, Prabhakaran et al. (2006) observed greater frontal activations for listeners to integrate low-compared to high-word frequency information when accessing a word from the lexicon during lexical decision tasks. In particular, Prabhakaran et al. (2006) observed greater activation in the left inferior frontal gyrus (LIFG) for low- as compared to high-frequency words. They suggest that this greater activation for low- as compared to high-frequency words is indicative of overall lower activation levels for low-frequency words that require greater cognitive resources to access from the lexicon as compared to high-frequency words.

Connectionist models also suggest that processing capacity *emerges* from the interaction between features inherent in the language input (e.g., frequency and regularity of patterns in the language) and innate biological–architectural factors of the individual speaker. These models show that individual differences in language processing are not due to differences in working memory capacity as traditionally interpreted but are the result of differences in the representational strength of the long-term linguistic knowledge being manipulated (e.g., Seidenberg and MacDonald, 1999; MacDonald and Christiansen, 2002). This suggests that the integration of various sources of information provided by lexical frequency determine the computational resources needed and thus the extent of neural activation in response to different computational demands.

Although the influence of cognitive effort inherent in word frequency has not been examined directly in verbal working memory research, this suggests that differences in the cognitive effort required to process high- versus low-frequency words may extend beyond short-term memory and the retrieval of items from the lexicon to include verbal working memory capacity and the influence that representational strength of words (i.e., activation thresholds) has on mental effort placed on the listener when having to simultaneously process, maintain in memory, and manipulate words during verbal working memory (Mainela-Arnold and Evans, 2005; Mainela-Arnold et al., 2010a, b). If one assumes that lexical representations in long-term memory and processing capacity are primitives that do not vary independently from each other and that the cognitive architecture of working memory capacity is not distinct from these mental representations, then differences in representational strength of words in the mental lexicon should directly influence cognitive processing load and verbal working memory capacity (Pearlmutter and MacDonald, 1995; MacDonald and Christiansen, 2002).

To examine directly the cognitive effort involved in encoding, maintaining, and updating spoken words differing in word frequency we use the classic *n-*back (*n* = 2) working memory paradigm. The main aim was to understand the neural correlates of the cognitive effort related to continuously updating information in working memory (Verhaeghen and Basak, 2005; Basak and Zelinski, 2013) under a fixed capacity. Using 2-back task, we could disentangle updating from capacity, since they are considered to be two separate indices of working memory (Basak and Verhaeghen, 2011a, b; Basak and Zelinski, 2013). Verbal 2-back fMRI tasks, albeit with visually presented information, engage greater connectivity of fronto-parietal brain network than tasks that do not require memory updating (O’Connell and Basak, 2018). To avoid the challenges inherent in conducting spoken word recognition tasks with fMRI, we instead used functional near-infrared spectroscopy (fNIRS). While behavioral accuracy and reaction times have been viewed as indirect measures of processing effort, non-invasive neuroimaging techniques such as fNIRS are well suited to directly measure changes in cognitive effort and mental work load in response to changes in task demands. fNIRS is an optical neuroimaging technique that measures cerebral hemodynamic changes associated with neuronal activities that offers several benefits over fMRI. In particular, NIRS systems have low sensitivity to motion artifacts and are portable, making them particularly suitable for spoken language studies that require quiet listening environments (Hoshi, 2007; Ferrari and Quaresima, 2012). In addition to whole-head montages, smaller fNIRS optode montages can be used to measure task-related localized activity, such as the prefrontal cortex, a particular strength for this study, since there is evidence that mental effort directly translates into hemodynamic changes in the prefrontal regions (Sato et al., 2013; Fishburn et al., 2014; Herff et al., 2014; Peck et al., 2014; Gateau et al., 2015; Yuksel et al., 2015). For example, Mandrick et al. (2016) examined the neural correlates of mental workload using a modified version of the standard *n*-back working memory paradigm and observed that fNIRS-measured hemodynamic responses in the prefrontal cortex directly corresponded to mental workload, with significantly greater changes being evident in both the left and right prefrontal cortex for high as compared to low cognitive demands.

In this study we ask if the cognitive effort required on the part of the listener to encode, update, and actively maintain spoken words in memory differs for high-frequency words as compared to low-frequency words. If higher frequency words have higher resting activation levels and lower access thresholds as suggested by short-term memory research, this should translate directly into lower mental effort required on the part of the listener to access these high-frequency words from the lexicon as evidenced by lower cortical activation in the prefrontal cortex. The null hypothesis to be tested is that there will be no difference in the cognitive effort required for the listener to simultaneously process, store, and manipulate high- versus low-frequency words during an *n-*back working memory tasks, with no difference being observed in behavioral measures (accuracy, reaction time) or prefrontal hemodynamic changes across the high- and low- word frequency conditions. Alternatively, the null hypothesis would be rejected if listeners’ behavioral accuracy is greater, speed of processing is faster, and hemodynamic activities in the prefrontal cortex are lower in the high word frequency condition as compared to the low word frequency condition.

## Materials and Methods

### Experimental Design

In this study we leveraged the experimental control provided by the *n*-back task to examine potential differences in mental effort required to hold high- versus low-frequency words in memory. Because the *n*-back task holds all verbal processing demands *except* storage constant, by holding *n* constant, it allows us to compare directly the processing demand that word frequency places on the listener’s ability to simultaneously process and encode incoming speech while holding information in memory. In the standard *n*-back paradigm, participants are presented with a continuous list of items and must determine if a given item is the same as an item *n* back in the sequence. For example, in the 2-back design participants determine if the target item is the same as the item that was 2-back in the sequence. It requires participants to actively maintain and update the working memory representation after each trial to keep track of the order of the last *n* items in the sequence (Owen et al., 2005; Jaeggi et al., 2010). In this study, participants completed a 2-back auditory working memory task where they heard a series of words presented one at a time and had to decide whether the word that they heard was the same word as they heard 2-back in the sequence. Participants were told to press the space bar on a keyboard as soon as they heard the same word. E-prime presented the stimuli and recorded participant’s behavioral responses including accuracy and reaction time (RT).

Participants completed a total of seven blocks of high word frequency words (the “HF” blocks) and seven blocks of low word frequency words (the “LF” blocks). Using a conventional fMRI block design, HF and LF blocks were presented in an alternating manner beginning with a HF block. Seven different fixed random-order sequences of the hits and correct rejections were created and assigned to each of the seven HF and LF blocks so that the serial order of hits/correct rejections did not differ across those blocks. The hit rate (25%) was the same for all of the blocks with no more than three consecutive hits occurring in a row. All blocks contained a total of 98 words. The initial phoneme of target words within each of the blocks was controlled so that initial phoneme of the congruent target/non-target pairs differed. The lists were also controlled so that the distribution of initial consonants in the blocks did not differ for the HF and LF lists. Word order in the lists was also constrained to ensure that there was no semantic priming effect between target/non-target words (word lists are provided in Supplementary Tables S1, S2).

Participants sat in front of a computer screen in a sound attenuated, dark room. Linked ear clips connected to insert earphones were placed in the participants’ ears and stimuli were presented binaurally. To ensure participants understood the task, participants were trained on 22 visual practice trials (clip art color pictures of common animals and objects (i.e., *pig, tree, stove, lamp*) and then 22 auditory trials, prior to starting the study. Feedback was given for each trial during the training period. Participants were required to complete all practice trials to the satisfaction of both the participant and the experimenter before continuing to the experimental task.

### Stimuli

The stimuli consisted of one-syllable real words. In order to insure findings were resultant from frequency, the words in the high-frequency (HF) and low-frequency (LF) conditions differed in both whole word and sublexical frequency: word frequency, *F*(1,13) = 310.5, *p* < 0.001, partial η^2^ = 0.353, and biphone phonotactic frequency, *F*(1,13) = 9877, *p* < 0.001. Words in the HF condition had both high whole word and sublexical phonotactic frequency. Words in the LF condition had both low whole word and sublexical phonotactic frequency because we hypothesized that words having both high frequency at the sublexical and whole word levels should have an additive effect of requiring less mental effort to encode, maintain and update in verbal working memory as compared words have low word and sublexical phonotactic frequency (Mainela-Arnold and Evans, 2005; Mainela-Arnold et al., 2010a, b). Other factors known to influence speed and accuracy of lexical processing such as neighborhood density and imageability were controlled across the two conditions (Walker and Hulme, 1999). The effect of phonotactic frequency is often masked by the inhibitory effect of neighborhood density, in part because these two measures are highly correlated and the correlation effect has not always been controlled for in studies (i.e., Pisoni et al., 1985; Frauenfelder et al., 1993; Coady et al., 2010). Specifically, words having high phonotactic frequency from large neighborhoods (i.e., neighborhood density) are processed *slower* than high phonotactic frequency words from smaller neighborhoods (Vitevitch and Luce, 1998, 1999; Dufour and Frauenfelder, 2010). Neighborhood density was low for both frequency conditions with all words having neighborhood density ratings at or above 20 and did not differ across the HF and LF conditions, *F*(1,12) = 0.01 and *p* = 0.92. Imageability ratings for all of the words was 5.0 or higher and also did not differ significantly for the HF and LF conditions, *F*(1,13) = 0.29 and *p* = 0.60. See Table 1.

**TABLE 1:** Word frequency, phonotactic probability, imageability, and neighborhood density for each block in the high frequency (HF) and low frequency (LF) conditions.

**Condition**	**Block**	**Word frequency^a^**	**Phonotactic probability^b^**	**Imageability^c^**	**Neighborhood density^d^**
HF	1	191.72	3.17	5.11	22.03
	2	182.65	3.12	5.30	21.51
	3	203.43	3.15	5.20	21.94
	4	176.68	3.14	5.25	21.00
	5	251.06	3.21	5.27	21.20
	6	215.89	3.14	5.31	20.75
	7	253.74	3.17	5.26	20.60
	Mean ± SD	210.74 ± 28.95	3.16 ± 0.03	5.24 ± 0.06	21.29 ± 0.52
LF	1	3.09	1.36	5.44	20.23
	2	2.43	1.26	5.13	21.67
	3	2.68	1.32	5.22	21.38
	4	2.47	1.27	5.17	20.66
	5	2.29	1.26	5.13	21.92
	6	2.20	1.25	5.22	21.09
	7	2.26	1.27	5.21	22.32
	Mean ± SD	2.49 ± 0.29^∗^	1.28 ± 0.04^∗^	5.22 ± 0.10^ns^	21.32 ± 0.67^ns^

*^*a*^MRC Psycholinguistic Database; http://www.psy.uwa.edu.au/mrcdatabase/uwa_mrc.htm. ^*b*^Vitevitch and Luce, 2004; http://www.bncdnet.ku.edu/cgi-bin/DEEC/post_ppc.vi. ^*c*^Washington University in St. Louis; http://128.252.27.56/Neighborhood/Home.asp. ^*d*^Cortese and Fugett, 2004, http://www.psychonomic.org/ARCHIVE/. ^∗^*p* < 0.001. ns = non-significant.*

Stimuli were digitally recorded, at 44.1 Hz, by an adult female native speaker of English. Each word file was 1,000 milliseconds with 50 milliseconds of silence at the beginning, and with each word production lasting approximately 700-millisecond long. Each sound file was trimmed to the appropriate length using Audacity. Each block was created from a 1,000-millisecond sound file with 50 milliseconds of silence at the beginning, a 5-millisecond envelope to the word in duration, with an interstimulus interval of 500 milliseconds. Each block lasted 90 s, and was separated from the next occurring block by 15 s of silence where the participants were instructed to rest and move as little as possible.

### Participants

A total of 25 college-aged adults (18–23 years; 20 females) from the University of Texas at Dallas participated in the study. All participants had normal language, were strongly right hand dominant, monolingual speakers of English. No participant was using psychotropic medications such as stimulants or anti-depressants. No participant had a history of neurological injury or disease, seizure disorder, or psychiatric diagnosis. All participants completed written informed consent protocols in accordance with the Declaration of Helsinki as well as the guidelines of the University of Texas at Dallas Institutional Review Board (IRB), which approved the protocol. Participants received financial compensation or college credit for their participation in the study.

### Functional Near Infrared Spectroscopy

**FIGURE 1:**
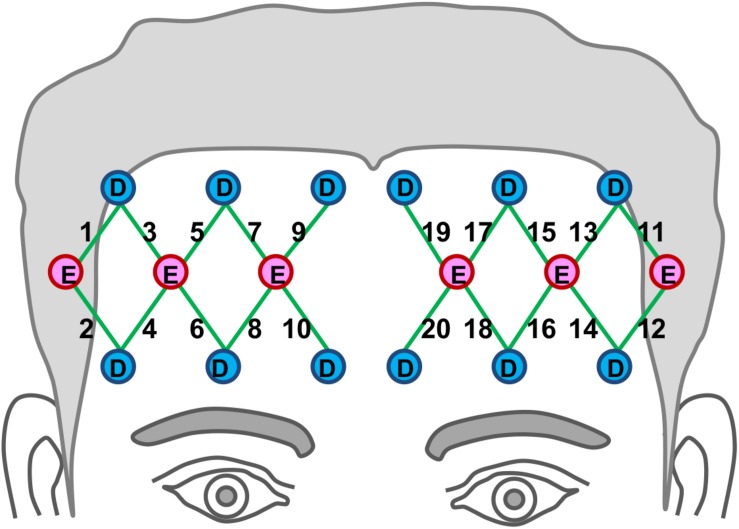
Location of fNIRS optodes on participants’ forehead. Emitters are marked as “E” in red circles and detectors are marked as “D” in the blue circles. Green lines represent the 20 channel paths.

A continuous-wave, multi-channel fNIRS system was used to acquire hemodynamic activities in the prefrontal region (TechEn, Inc., Milford, MA, United States). The system uses near-infrared lasers at 690 and 830 nm as emitters, and avalanche photodiodes (APDs) as detectors. A fiber optic probe that contained six emitting optodes and 12 detecting optodes was place bilaterally and symmetrically on the participant’s forehead, as shown in Figure 1. The probe was secured in place with an elastic band during the experiment. The bottom of the probe array was placed just above the eyebrows. This optode montage provides 20 channels of hemodynamic measurements at a constant emitter-detector distance of 3 cm. Areas underlying the channels are approximately over Brodmann’s areas 10 and 46. The data sampling rate was 25 Hz.

### Data Processing and Artifact Removal

The fNIRS data were processed using Homer software (Huppert et al., 2009). The raw data were band-pass filtered between 0.005–0.2 Hz to remove slow drifts and high-frequency noises, and then converted into changes in optical density. Blocks corrupted by large motion artifacts (e.g., yawns, coughs) were manually removed. Changes in oxygenated hemoglobin (HbO_2_) and deoxygenated hemoglobin (Hb) concentrations were calculated based on the Modified Beer-Lambert Law with a partial path-length factor of 6.0 for both wavelengths. At last, block-averaged HbO_2_ and Hb responses for each of the 20 channels were derived from the motion-free blocks, which were then used to reconstruct topographic images of prefrontal activation based on default Homer functions. After all the steps in data processing were completed, the data from four subjects was deemed too noisy and these participants were removed from analysis post cleaning. Subsequent analysis was conducted on the behavioral and hemodynamic data for the remaining 21 participants (18 female).

### Statistical Analysis

For the behavioral analysis, data were corrected using the Greenhouse-Geisser correction applied to the probability values to adjust for repeated measures. To address the potential problem of measures of accuracy such as percent correct where the individual’s sensitivity to correct responses is confounded with biases to select (or avoid) particular stimuli, *d-prime* (*d’*) was used instead of percent correct. *d’* was calculated from the average hit and false accept rates (MacMillan and Kaplan, 1985) for all trials in each block to provide a measure of the participant’s ability to recognize a target match. *d’* sensitivity index in *z* units is used in signal detection theory to assess performance. In contrast to proportion correct that is affected by the listener’s sensitivity and inherent response bias, the *d’* statistic differentiates the means for the signal (i.e., target) versus noise (i.e., foils) and is a direct measure of the listener’s ability to recognize/differentiate target from foil trials. In this study, higher *d’* values indicate that the participant was better able to perform the task - was more accurate in recognizing the match for those trials where the word was the same as that 2-back in the sequence (fewer misses or false alarms). A *d’* value above zero indicates that the participant was able to recognize the match in the target with the word 2 back in the sequence. For this study specifically, a *d’* of 8.6 corresponds to 100% accurate performance. Reaction times were analyzed for correct trials only with no trimming of outliers. Repeated measures ANOVAs were conducted for *d*’ and (RT) with word frequency (HF, LF) as the within subject factor.

For the hemodynamic analysis, data were corrected using the Greenhouse-Geisser correction applied to the probability values to adjust for repeated measures. A repeated measures ANOVA was conducted examining channel-wise hemodynamic change across the prefrontal cortex in response to word frequency (Tian et al., 2014). Channel-wise mean hemodynamic changes were calculated for each subject and compared for the HF and LF conditions for each participant. Then separate *t*-tests were run to identify regions of interest (ROIs) comparing: (1) difference in hemoglobin levels for HF vs. LF at each channel, (2) difference in hemoglobin levels for HF versus resting state at each channel, and (3) difference in hemoglobin levels for LF versus resting state at each channel. Both *t*- and *p*-statistic values were derived from the *t*-tests for each channel for each of these comparisons. Due to the inherent diffusive characteristics of fNIRS imaging, channels that were next to each other spatially were collapsed into a single ROI.

To determine if the hemodynamic responses were related to participants’ performance, bivariate correlation analyses were conducted between behavioral measures (*d’* and RT) and mean hemodynamic changes within the ROIs identified in each of the three comparison conditions.

## Results

### Behavioral Scores

**FIGURE 2:**
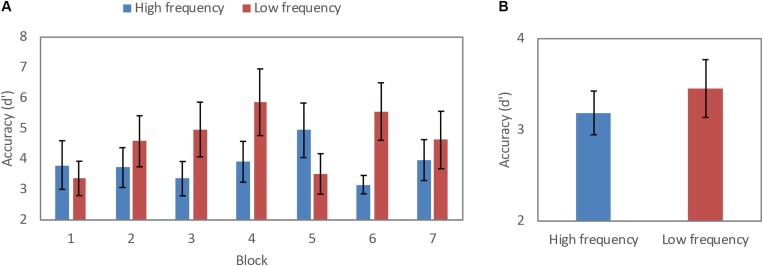
Accuracy in *d*-prime for high- and low-frequency across **(A)** blocks, and **(B)** condition (error bars: 95% confidence interval).

Accuracy as measured by *d’* for the HF and LF blocks is shown in Figure 2. A repeated measures ANOVA revealed a significant main effect of word frequency, *F*(1,20) = 13.20, *MSE* = 38.02, *p* < 0.01, partial η^2^ 0.40, power 0.93, where participant’s accuracy in correctly detecting the target word 2-back in the sequence was better in LF (*M* = 4.64) as compared to the HF condition (*M* = 3.84). For the main effect of block, both the linear effect, *F*(4.48,89.6) = 2.61, *MSE* = 8.31, *p* < 0.05, partial η^2^ 0.12, power 0.75 and quadratic effect, *F*(1,20) = 6.16, *MSE* = 16.63, *p* < 0.05, partial η^2^ 0.24, power 0.66 were significant, indicating that participants’ performance accuracy increased significantly until the middle set of blocks and then declined slightly for the final set of blocks in the experiment. There was also a significant interaction effect of frequency by block, *F*(4.45,89) = 7.82, *MSE* = 22.75, *p* < 0.01, partial η^2^ 0.28, power 0.99, where there was an overall greater increase in accuracy across the LF blocks over the course of the experiment as compared to the HF blocks.

**FIGURE 3:**
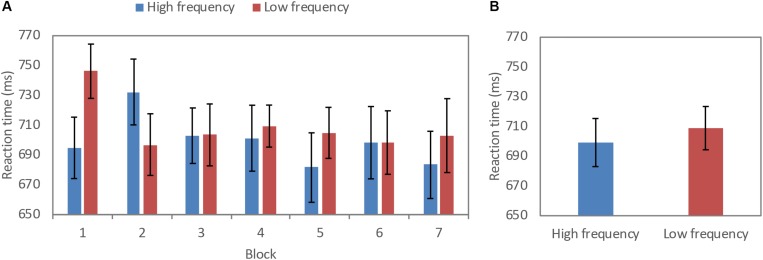
Reaction time (millisecond) for high frequency and low frequency across **(A)** blocks, and **(B)** condition (error bars: 95% confidence interval).

RTs for correct trials are shown in Figure 3. A repeated measures ANOVA revealed a significant main effect of word frequency, *F*(1,20) = 13.38, *MSE* 7508.46, *p* < 0.01, partial η^2^ 0.40, power 0.94, where RTs for correct trials were significantly faster for the HF (*M* = 699.19 ms) as compared to the LF condition (*M* = 708.87 ms). There was a main effect for block, where RTs were faster for in both HF and LF blocks over the course of the task, *F*(3.65,73.03) = 2.59 and, *MSE* = 7798.72, *p* < 0.05, partial η^2^ 0.12, power 0.67. There was also a significant frequency by block interaction, *F*(2.8,56.01) = 6.70, *MSE* = 14792.29, *p* < 0.01, partial η^2^ 0.25, power 0.96, where that pattern of RTs became faster over the course of the entire blocks was significantly greater over the LF blocks as compared to HF blocks.

### Hemodynamic Changes in the Prefrontal Cortex

**FIGURE 4:**
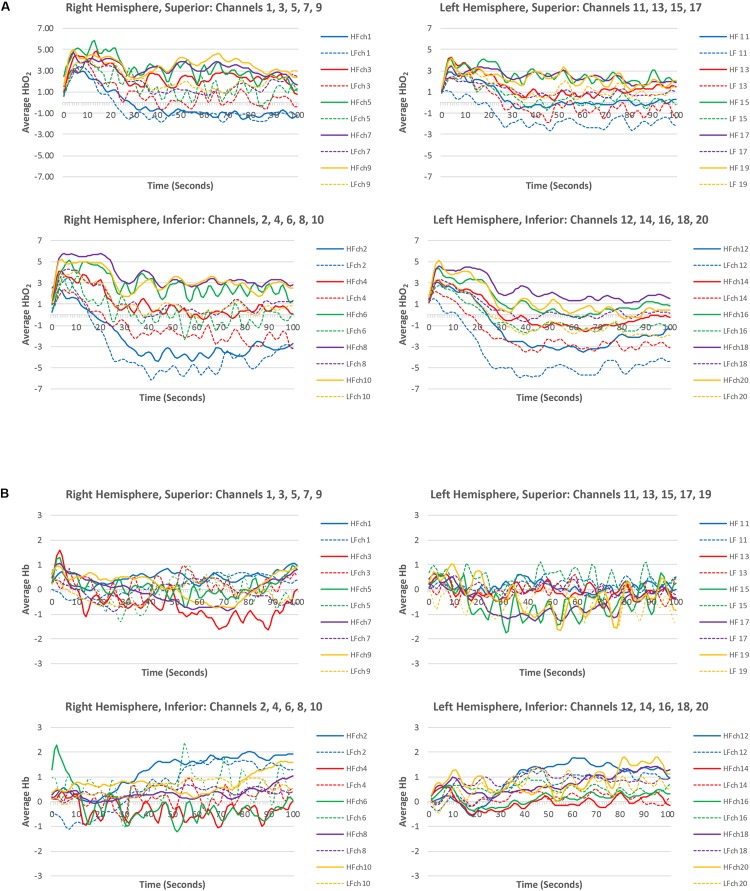
Channel-wise, block-averaged levels of **(A)** HbO_2_ and **(B)** Hb, for high-frequency and low-frequency conditions prior to statistical analysis.

Channel-wise, block-averaged hemodynamic changes across the course of HF and LF conditions are shown in Figure 4. ANOVA analysis revealed a significant main effect of word frequency *F*(1,20) = 18.16, *MSE* = 31.70, *p* < 0.01, partial η^2^ 0.48, power 0.98, for HbO_2_, where HbO_2_ was significantly greater for HF (*M* = 1.69) as compared to LF (*M* = −0.04) words. In contrast, no significant main effect of frequency was found for Hb, *F*(1,20) = 0.14, *MSE* = 0.10, *p* = 0.72, partial η^2^ < 0.01, power = 0.06.

**FIGURE 5:**
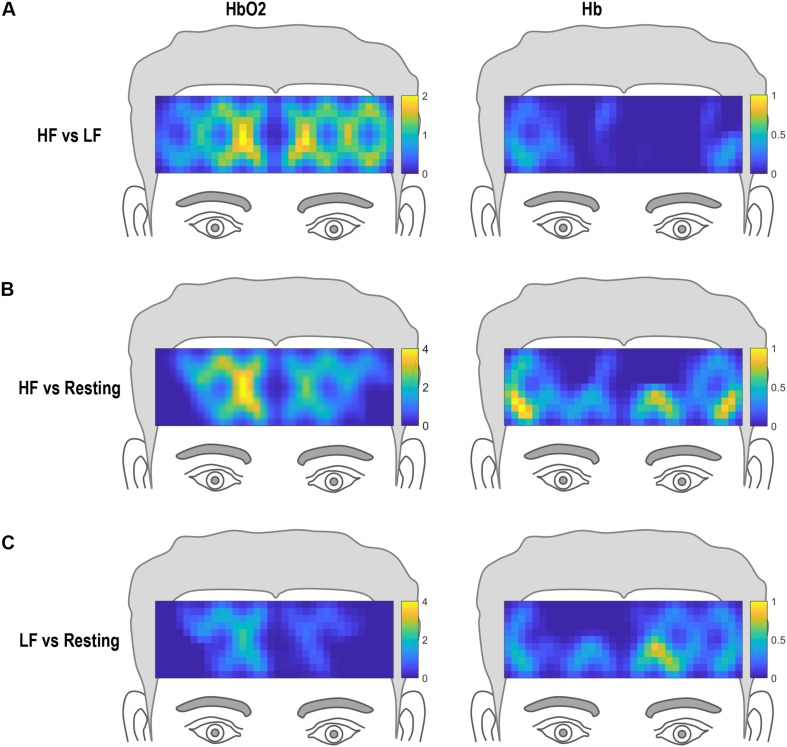
Hemodynamic topography of activation in the prefrontal cortex. **(A)** HbO_2_ and Hb topography for high frequency (HF) versus low frequency (LF) conditions, **(B)** HbO_2_ and Hb topography for high frequency (HF) versus resting state, and **(C)** HbO_2_ and Hb topography for low frequency (LF) versus resting state.

The hemodynamic topography of the prefrontal cortex for HF vs. LF, HF versus resting state, and LF versus resting state are shown in Figures 5A–C for HbO_2_. Statistical analysis (*t*- and *p*- values) of hemodynamic changes across the three different comparisons for each channel are summarized in Table 2. For HF versus LF comparison, the hemodynamic activity differed significantly in 11 of the 20 channels, characterized by greater increase of HbO_2_ in HF as compared to LF. No differences were observed between HF and LF conditions for Hb changes.

**TABLE 2:** HbO2 and Hb statistics (*t* and *p*) for high frequency (HF) versus low frequency (LF), HF versus resting, and LF versus resting conditions channel HF vs. LF HF vs. resting LF vs. resting.

**Channel**	**HF vs. LF**				**HF vs. Resting**				**LF vs. Resting**			
			
	**HbO2**		**Hb**		**HbO2**		**Hb**		**Hb02**		**Hb**	
						
	***t***	***p***	***t***	***p***	***t***	***p***	***t***	***p***	***t***	***p***	***t***	***p***
1	–0.40	0.69	1.16	0.26	–0.82	0.42	1.38	0.18	–0.83	0.42	0.70	0.49
2	0.11	0.91	1.51	0.15	−2.60^∗^	0.02	2.89^∗∗^	0.01	–3.48^∗∗^	0.00	1.98	0.06
3	1.82	0.08	–0.80	0.43	2.13^∗^	0.04	–0.66	0.52	0.76	0.45	0.69	0.50
4	1.68	0.11	–0.71	0.49	0.35	0.73	–0.24	0.81	–1.43	0.17	1.07	0.30
5	1.93	0.07	0.56	0.58	3.02^∗∗^	0.01	0.49	0.63	1.30	0.21	–0.32	0.75
6	1.87	0.07	–0.83	0.41	1.34	0.20	–0.17	0.86	–0.10	0.92	1.22	0.23
7	2.00	0.06	0.11	0.91	3.89^∗∗^	0.00	–0.14	0.89	1.91	0.07	–0.32	0.75
8	3.24^∗∗^	0.00	–0.73	0.48	2.86^∗∗^	0.01	0.94	0.36	0.81	0.43	1.57	0.13
9	1.90	0.07	0.33	0.75	3.64^∗∗^	0.00	0.47	0.65	2.34^∗^	0.03	0.00	1.00
10	3.07^∗∗^	0.01	–0.05	0.96	2.67^∗∗^	0.01	1.43	0.17	0.92	0.37	2.35^∗^	0.03
11	2.14^∗^	0.04	–0.64	0.53	0.74	0.47	0.19	0.85	–1.54	0.14	0.75	0.46
12	2.49^∗^	0.02	0.14	0.89	–1.93	0.07	1.21	0.24	–3.02^∗∗^	0.01	1.70	0.10
13	2.44^∗^	0.02	–0.09	0.93	1.72	0.10	–0.04	0.97	–0.26	0.80	0.11	0.92
14	2.62^∗^	0.02	–0.38	0.71	0.06	0.96	0.01	0.99	–1.76	0.09	0.49	0.63
15	2.78^∗∗^	0.01	–1.01	0.32	2.47^∗^	0.02	–0.68	0.51	0.69	0.50	1.09	0.29
16	3.17^∗∗^	0.00	–1.19	0.25	1.69	0.11	0.39	0.70	–0.31	0.76	1.48	0.15
17	2.57^∗^	0.02	–1.92	0.07	3.39^∗∗^	0.00	–1.64	0.11	1.85	0.08	0.21	0.84
18	3.50^∗∗^	0.00	–0.54	0.60	2.65^∗∗^	0.01	1.23	0.23	0.86	0.40	2.65^∗∗^	0.01
19	1.51	0.14	0.24	0.82	3.23^∗∗^	0.00	–1.03	0.32	2.19^∗^	0.04	–0.73	0.47
20	2.53^∗^	0.02	–0.25	0.80	1.27	0.22	1.35	0.19	–0.04	0.97	2.36^∗^	0.03

*^∗^*p* < 0.05, *^∗∗^p* ≤ 0.01.*

Compared to resting state, HF words evoked robust widespread HbO_2_ changes across both left and right hemispheres (Figure 5B). This pattern of activation was significant in 11 of the total 20 channels (Table 2). Hb changes were also evident, with significant changes from resting state seen in 1 of the 20 channels in the right inferior region overlapping the homologous region of significant decrease in HbO_2_ response. For the LF condition compared to resting state, a bilateral response was seen for both HbO_2_ and Hb (Figure 5C). For HbO_2_, differences in activation from rest were significant in 4 of the 20 channels. For Hb, differences in activation for LF versus rest were seen in 3 of the 20 channels (Table 2).

### Correlations Between Behavioral and Hemodynamic Measures

**FIGURE 6:**
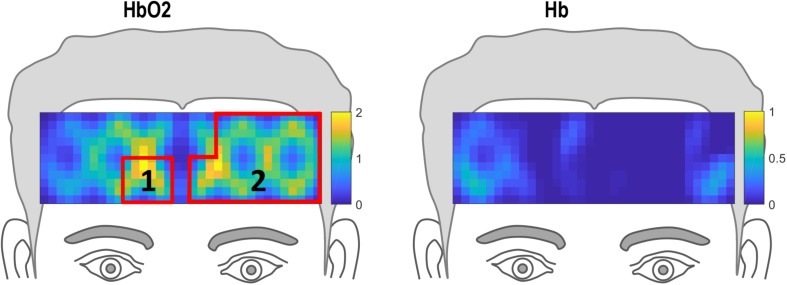
Regions of interests (ROIs) for high frequency (HF) compared to low frequency (LF) defined by clusters of channels where hemodynamic activation differed significantly for HF compared to LF.

The ROIs for the HF vs. LF comparison are shown in Figure 6. The correlational analyses for this comparison are shown in Table 3. For the HF versus LF comparison, there was no significant correlation between hemodynamic changes and behavioral scores (*d’* and RT), indicating that there was no relationship between participants’ behavioral performance in the *n-*back working memory task and prefrontal HbO_2_ activation levels based on word frequency.

**TABLE 3:** Bivariate correlations for *d’*, RT and ROIs 1 and 2 shown in Figure 6 for HF versus LF comparison.

	**Channel**		***R***	
ROI 1	8,10	*d’*	−0.26	*p* = *0.26*
		RT	0.12	*p* = *0.61*
ROI 2	11,13,15,17,12,14,16,18,20	*d’*	−0.10	*p* = 0.66
		RT	0.31	*p* = 0.18

**FIGURE 7:**
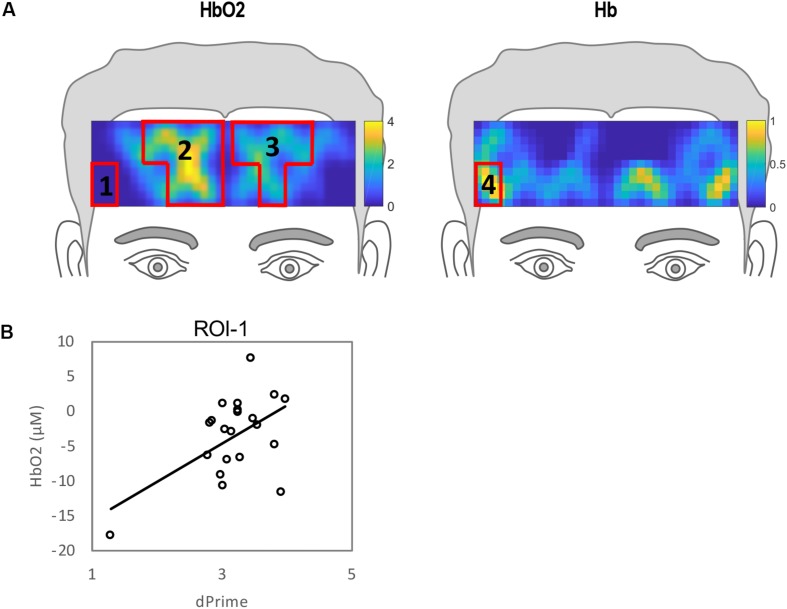
**(A)** Regions of interests (ROIs) for high frequency compared to resting state defined by clusters of channels where hemodynamic activation differed significantly for HF versus resting. **(B)** Correlation between individual performance accuracy and hemodynamic responses to ROI 1.

The ROIs for HF vs. resting state are shown in Figure 7. The correlational analyses for this condition are shown in Table 4. It is noted that both HbO_2_ and Hb results indicate a significant change in the corner of the right hemisphere (ROI 1 and ROI 4), however, only the hemodynamic signal within ROI 1 was significantly correlated with *d’* (*R* = *0.54*, *p* < 0.01) indicating that participants’ accuracy for HF trials was related to degree of HbO_2_ changes in the lateral inferior region of the right hemisphere. No significant correlation was found in the other three ROIs.

**TABLE 4:** Bivariate correlations for *d’*, RT and ROIs 1, 2, and 3 shown in Figure 7 for high frequency versus resting state.

	**Channel**		***R***	
ROI 1	2 (HbO2)	*d’*	0.59^∗^	*p* = *0.005*
		RT	–0.24	*p* = *0.29*
ROI 2	3,5,7,9,8,10	*d’*	0.34	*p* = *0.13*
		RT	–0.05	*p* = *0.82*
ROI 3	15,17,19,18	*d’*	0.12	*p* = *0.62*
		RT	0.08	*p* = *0.73*
ROI 4	2 (Hb)	*d’*	–0.06	*p* = *0.79*
		RT	0.11	*p* = *0.64*

*^∗^p = 0.005.*

**FIGURE 8:**
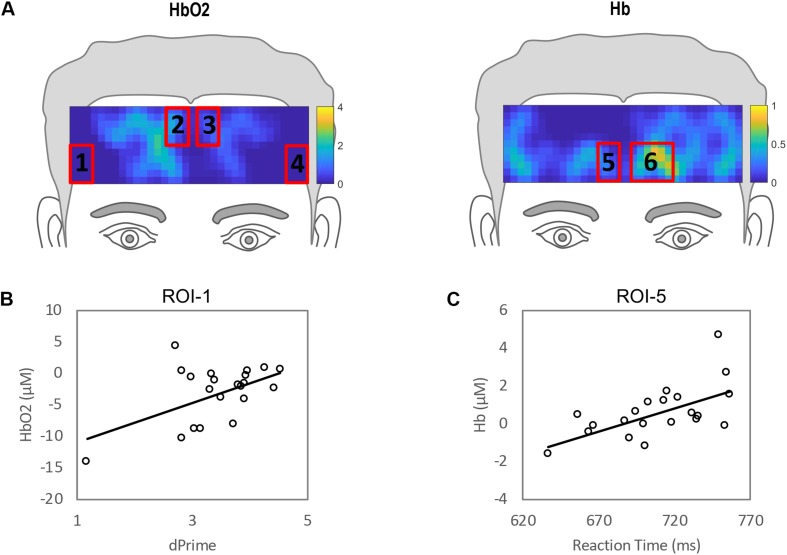
Regions of interests (ROIs) for Low Frequency compared to resting state defined by clusters of channels where hemodynamic activation differed significantly different for LF versus resting. **(B)** Correlation between individual performance accuracy and hemodynamic responses to ROI 1. **(C)** Correlation between individual reaction time and hemodynamic responses to ROI 5.

The ROIs for LF vs. resting state are shown in Figure 8. The correlational analyses for this condition are shown in Table 5. The HbO_2_ changes were significantly correlated with *d’* in ROI 1 (*R* = 0.52, *p* < 0.02). The Hb changes were significantly correlated with RT in ROI 5 (*R* = 0.61, *p* < 0.01).

**TABLE 5:** Bivariate correlations for *d’*, RT and ROIs 1–6 shown in Figure 8 for low frequency vs. resting state.

	**Channel**		***R***	
ROI 1	2	*d’*	0.52	*p* = 0.02
		RT	–0.03	*p* = 0.89
ROI 2	9	*d’*	0.30	*p* = 0.18
		RT	–0.21	*p* = 0.34
ROI 3	19	*d’*	0.03	*p* = 0.91
		RT	0.11	*p* = 0.62
ROI 4	12	*d’*	0.17	*p* = 0.46
		RT	0.21	*p* = 0.35
ROI 5	10	*d’*	–0.13	*p* = 0.56
		RT	0.61^∗∗^	*p* = 0.003
ROI 6	18,20	*d’*	–0.02	*p* = 0.92
		RT	0.33	*p* = 0.15

*^∗∗^p = 0.003.*

## Discussion

In this study, the first to directly examine the cognitive effort required for listeners to processing spoken words during a complex working memory updating task, we asked if spoken words differing in frequency of occurrence also differed in the mental effort required by the listener to hold in verbal working memory. To date, spoken word frequency has been investigated primarily in serial recall tasks that predominantly tap short term working memory as they require the listener to simply maintain lists of words. Real time sentence language comprehension, in contrast, requires listeners to rely on complex verbal working memory which includes not only the encoding, maintaining, and reactivating of words in memory but also the simultaneous inhibition of lexical cohort competitors and updating the context based on subsequent words/phrases. In this study, we used 2-back task to examine the cognitive effort of spoken word frequency in complex working memory. Listeners were required to hold a series of spoken words in memory while simultaneously determining if each word in the sequence was a match to a word having occurred two back in the sequence.

Historically, the focus of studies investigating word frequency and hemodynamic changes in brain activity has been on lateral language network related brain regions. In this study we focused instead on changes in HbO_2_ and Hb hemodynamic activation levels in the prefrontal lobe to examine potential differences in the mental effort require by the listener to words differing in word frequency in complex working memory. Our hypothesis was that differences in the strength of long-term associative links inherent in the frequency of spoken words would translate into differences in the mental effort required on the part of the listener to encode and maintain information in working memory as evidenced by *lower* HbO_2_ hemodynamic activation in the frontal regions for high- as compared to low-frequency words.

In this study we observed differences in hemodynamic activation for listeners when processing high- as compared to low-frequency words in complex working memory, but contrary to our expectations, the hemoglobin changes were *opposite* our expectations. Specifically, we observed HbO_2_ levels to be *higher* for high frequency words as compared to low frequency words, and behaviorally, participants’ accuracy performance was *poorer* for the high- as compared to low-frequency word conditions. The hemodynamic activation in the frontal lobe mirrored participants’ performance, with hemodynamic activation being significantly correlated with accuracy in the most lateral inferior probes in the right hemisphere when processing high and low-frequency words.

Unlike simple serial recall tasks, in this study participants were required to hold a sequence of words in memory while simultaneously maintaining lexical activations while updating incoming information and detecting a match with a target word 2-back in the sequence. Previous studies reporting better accuracy and faster processing speed for high- as compared to low-frequency words have used other types of tasks, such as serial recall, which require the use of only a portion of working memory, short-term memory, and not a larger network in the working memory system that required dynamic updating. Moreover, the current task is a item-recognition paradigm, whereas previous studies used recall. The effect of word frequency is highly dependent on the type of LTM paradigm implemented, recognition vs. recall. The unique and memorable aspects of low frequency words support better recognition than high frequency words (Diana and Reder, 2006) but high frequency words have greater recall than low frequency words, because the contextual cues support greater elaboration in high frequency words due to regularity of use (Gregg et al., 1980; Gillund and Shiffrin, 1984; MacLeod and Kampe, 1996). While this previous work has provided the basis for the idea that high-frequency words have been presumed to have higher resting activation thresholds for recognition, clearly our data suggest that there may be a speed-accuracy trade off: the participants were more accurate at recognizing target words 2-back in the sequence for low-frequency words as compared to high-frequency words; on the other hand, they were quicker to correctly recognize that the current word was a match to the target word 2-back in the sequence for high-frequency words.

We measured accuracy using *d’* a statistic used in signal detection theory that provides a combined measure of listener sensitivity for discriminating a signal from noise while controlling for listener response bias. For each word in the sequence there were 4 options. If the current word was a match to the target word 2 back in the sequence, the listener either responded correctly (HIT) or did not respond (MISS). In contrast, if the current word was *not* a match to the target work 2 back in the sequence, the listener either responded incorrectly (False Accept) or did not respond (correct rejection). Our data show that listeners where better able to respond correctly on those trials having a target matching word and inhibit a response on those trials having a non-target matching word when the words were low frequency. We initially thought that words having higher resting state activation thresholds and low access thresholds (i.e., high frequency) would be easier for the listeners to process. The data from this study suggest instead that the process of simultaneously holding in memory and matching words that have lower resting state activation levels and high access thresholds (i.e., low frequency) may have been easier for the participants to process. This may be a potential protective effect of low-frequency words in that the higher access thresholds required to “reactivate” the target word prevented listeners from inadvertently responding incorrectly. However, this protective effect of higher access thresholds resulted in an overall slower activation of the motor response once the listener “recognized” that the word matched the target 2-back in the sequence as compared to high-frequency words. Because the words in the high- and low-frequency conditions were matched on neighborhood density (all words in both conditions having low neighborhood density), possible confounding differences in the effects of neighborhood density did not come into play in this study.

The pattern observed from hemodynamic activations across the two conditions suggests that the effect of different access threshold levels for high- versus low-frequency words plays out in a different manner for complex working memory tasks where the listener must hold a sequence of words in memory while simultaneously checking to see if each incoming word is a match to a word just heard 2-back in the sequence. The data from this study suggest that complex working memory tasks such as these may recruit multiple brain systems beyond memory that are necessary for lexical activation, focused attention, and inhibition of lexical interference effects.

In this study, greater hemodynamic activation was observed extensively in bilateral frontal regions in the high-frequency condition. Prior work shows that anterior neural structures such as the middle frontal gyrus are implicated in executive control involving the maintenance and manipulation of information in working memory (D’Esposito et al., 2000) while activation of the LIFG emerges under conditions requiring selection among competing alternatives (Goldman-Rakic, 1994; Petrides, 1994; Courtney et al., 1997; Owen, 1997; Thompson-Schill et al., 1997). Similarly, Prabhakaran et al. (2006) found that the left supramarginal gyrus is engaged primarily in the process of activating phonologically similar lexical neighborhoods, but once the phonetic pattern of the word was activated, frontal regions are recruited during the controlled processing of the lexical items. Most importantly for this study, increased activation in the dorsal lateral prefrontal cortex (DLPFC) is highly implicated in the maintenance of information during a memory task (Dolcos et al., 2007). Moreover, increased connectivity of fronto-parietal network, which includes DLPFC, increases with cognitive effort from just maintaining and selecting between two tasks to maintaining, selecting and updating two sets of information (O’Connell and Basak, 2018). The higher HbO_2_ levels in this study suggest that words having higher resting state activation levels and low access thresholds (i.e., high frequency) may require greater executive control during complex working memory tasks that place higher demands during both maintenance and manipulation of verbal information.

The limited hemodynamic activation occurring for the low-frequency words adds evidence to these words having low resting state activation levels/high access thresholds. As mentioned for the high-frequency condition, greater activation of frontal regions has been reported under conditions requiring selection among competing alternatives (Thompson-Schill et al., 1997; Nozari and Thompson-Schill, 2016) suggesting that low-frequency words may have less competitors to choose between, thus reflecting the low HbO_2_ levels as compared to high-frequency words that have more competitors for the listener to select among and/or inhibit.

The greater difference between activation patterns between high- and low-frequency words for the left as compared to right prefrontal cortex may be due to the task being verbal and not visual in nature. Studies show the right hemisphere activates more robustly to visual information than for linguistic information (Levy and Wagner, 2011), whereas left ventrolateral prefrontal cortex (VLPFC) critically activates for tasks demanding access to, and the evaluation of, semantic information (Badre et al., 2005; Badre and Wagner, 2007). Therefore, one would expect the left hemisphere to react more dramatically between the word conditions in the left hemisphere as compared to the right, which is what we found. This study shows just how dramatic the role of extant knowledge is in influencing cognitive effort in working memory tasks. This also suggests that lack of control of extant knowledge in participants may be the reason for the vast discrepancy in results between imaging and behavioral studies of working memory in the literature.

We found correlation between increased levels of HbO_2_ in the right lateral areas and greater behavioral accuracy, an area that corresponds closely to the VLPFC. Interestingly, Levy and Wagner (2011) also found that VLPFC responds to decision-level conflicts/uncertainty, i.e., inhibition of extraneous, non-task related information (Dolcos et al., 2007; Kim et al., 2015). This explanation of VLPFC activation pattern would closely mirror our results for both word frequency conditions in that the greater inhibitory response, or the greater HbO_2_ changes, the less conflicting information that could interfere in the maintenance of information in working memory, thus resulting in improved accuracy.

There are certain limitations to this study that need mentioning. First, the use of NIRS only allows the general localization of activation. Second, because the focus of our study was prefrontal activation, changes in activation in language networks (e.g., superior temporal cortex, IFG, etc.) may have influenced the activation patterns observed in our study, but the optode array was not designed to examine these laterally based ROIs. For example, while low-frequency words had lesser activation in the left prefrontal cortex as compared to high-frequency words, it is possible that low frequency words may have had greater activation in more lateral temporal/parietal language ROIs as compared to high frequency words. Alternatively, we might have observed greater changes in activation in the prefrontal relative to changes in activation in left temporal lobes for high frequency as compared to low frequency words. Additionally, behavioral data was collected on “hits” only, as opposed to “hits” and “correct rejections” thereby limiting our fNIRS analysis to frequency averaged values for each participant, preventing us from investigate the impact of word frequency on cognitive load in a more refined manner. Using a design where participants are required to press one button when the word is a match and a second button when the word is not a match would allow future researchers to examine the different aspects of complex working memory in more detail.

A second concern with the design of this study is the absence of additional measures of focus of attention. Due to the design of the current study, we were unable to examine changes in hemodynamic activity during the time course of each block and over the course of all the blocks in the task. One possibility is that the HbO_2_ and Hb changes observed in this study instead reflect changes in focus of attention possibly due to listener fatigue over the course of the study. While we do not have direct measures of focus of attention, the behavioral data suggests participants were able to focus on the task at hand, as we observed better performance both in terms of accuracy and RTs both within and across the high- and low-frequency blocks. Although it was not the focus of the current study, future studies could extend our findings by studying the combined effects of capacity and updating with variable *n*.

Taken together the results of this study shows that amount of cognitive effort required for working memory tasks does not exist independent of extant knowledge, supporting embedded and connectionist models of working memory. Additionally, this study indicates that findings on short-term memory cannot be simply extended to more complex working memory tasks if models of working memory are used that do not include the interplay between working memory and extant knowledge. Future research should investigate how the different features of information impact cognitive effort requirements for working memory in all types of modalities.

## Data Availability Statement

The datasets generated for this study are available on request to the corresponding author.

## Ethics Statement

All participants completed the written informed consent protocols in accordance with the Declaration of Helsinki as well as the guidelines of the University of Texas at Dallas Institutional Review Board (IRB), which approved the protocol.

## Author Contributions

JE and FT designed the experiment. JE developed the stimuli. FT, AB-B, and JE collected the fNIRS data. FT, AB-B, and JE processed and analyzed the data. AB-B and JE wrote and edited the manuscript with input from FT and CB.

## Conflict of Interest

The authors declare that the research was conducted in the absence of any commercial or financial relationships that could be construed as a potential conflict of interest.
