# Supplementary material for: Word Frequency Is Associated With Cognitive Effort During Verbal Working Memory: A Functional Near Infrared Spectroscopy (fNIRS) Study
Source: Front Hum Neurosci. 2019 Dec 13;13:433. doi: 10.3389/fnhum.2019.00433 (PMC6923201; doi:10.3389/fnhum.2019.00433)
Supplement: Supplementary file 1 [file Data_Sheet_1.ZIP › Supplementary Material Presentation/Supplementary table 2.docx]

| High List 1 | High List 2 | High List 3 | High List 4 | High List 5 | High List 6 | High List 7 |
| --- | --- | --- | --- | --- | --- | --- |
| cup | ran | work | leg | dust | back | back |
| talk | girl | talk | bill | work | cup | cup |
| cup | cup | work | point | door | back | back |
| door | back | door | bill | gold | cup | rose |
| face | cup | moon | girl | face | men | back |
| rich | west | gold | cut | land | cup | moon |
| face | date | life | girl | feed | leg | back |
| rich | west | face | point | car | shop | land |
| face | bed | point | big | face | leg | team |
| pick | line | face | point | game | team | shop |
| moon | win | run | mass | car | leg | team |
| pick | fire | gas | road | march | west | shop |
| reach | moon | mold | mass | pool | life | ball |
| met | name | big | point | road | west | land |
| reach | land | life | rich | sat | dust | game |
| note | march | gold | big | ball | pool | curve |
| life | feed | life | sit | sat | dust | game |
| note | march | pick | big | ball | land | coat |
| point | look | moon | team | work | coat | white |
| note | win | pick | ship | ball | land | feed |
| fire | sat | curve | case | face | coat | white |
| call | rich | rest | moon | ball | fig | bill |
| fire | sat | curve | car | land | run | game |
| call | point | shop | moon | mold | fig | pace |
| fire | sat | road | big | land | run | land |
| run | goal | note | coat | moon | cup | fig |
| sat | sat | sat | side | part | note | man |
| mold | goal | note | kid | life | deep | pick |
| big | curve | sat | side | part | white | red |
| road | red | man | land | date | fig | gas |
| moon | deep | light | side | part | white | top |
| cut | goal | cut | mass | date | pull | gas |
| rest | door | type | case | pool | date | red |
| cut | west | cut | rose | back | top | curve |
| shop | light | wall | light | man | date | man |
| cut | back | dust | ship | dust | gas | dust |
| list | coat | wall | light | back | put | man |
| man | face | cup | team | dust | gas | dust |
| light | dust | wall | side | year | mold | man |
| wall | ship | lead | fire | face | win | dust |
| type | pool | met | name | land | ship | man |
| wall | ship | pool | coat | pick | look | fig |
| girl | door | ten | name | road | bed | case |
| dust | big | note | coat | pick | man | deep |
| girl | white | ten | gold | ship | bed | case |
| cup | team | date | list | top | curve | fig |
| lead | white | kid | gold | road | face | moon |
| met | deep | list | list | mass | reach | fig |
| pool | ten | kid | ran | back | call | moon |
| note | list | list | curve | mass | win | fig |
| work | ten | pull | ran | work | call | moon |
| list | met | base | lead | mass | team | leg |
| work | book | cold | pool | ship | north | moon |
| list | fig | dust | name | mass | team | year |
| date | book | cold | pass | land | ship | deep |
| top | gold | game | girl | nose | gold | pick |
| west | land | moon | board | feed | ship | fire |
| case | wide | cut | girl | book | car | base |
| game | mold | gas | book | goal | ship | life |
| met | wide | rose | gold | book | look | mass |
| past | mold | gas | book | cold | man | life |
| light | pace | rose | gold | book | base | deep |
| near | man | light | book | lead | gold | game |
| cold | pace | test | gold | dust | sat | kid |
| book | test | near | met | lead | life | lead |
| cold | board | test | gas | fire | fig | cup |
| book | mass | wall | march | lead | life | goal |
| part | board | part | cup | face | bill | fig |
| rose | ten | wall | point | bed | life | name |
| ten | game | ten | door | moon | top | mold |
| rose | bill | wall | point | nose | pace | name |
| wall | game | wall | feed | car | note | pace |
| top | coat | date | north | nose | back | life |
| sit | game | top | coat | car | name | fire |
| west | coat | date | win | kid | rose | path |
| reach | ship | cup | coat | pick | ball | fire |
| pace | coat | date | short | leg | fire | march |
| test | ship | nose | rich | pick | run | game |
| march | mold | reach | talk | deep | book | march |
| test | fire | mass | leg | fig | sat | point |
| point | mold | pace | date | coat | march | road |
| gold | car | mass | top | fig | bill | nose |
| court | face | white | date | back | march | Sit |
| wall | type | point | sat | fig | coat | top |
| pick | rich | gold | date | work | march | door |
| wall | moon | court | pass | met | door | met |
| pick | rich | gold | type | work | nose | door |
| mass | nose | back | pass | part | door | ship |
| fig | fig | fig | gold | feed | test | pace |
| mass | list | back | mass | dust | point | rich |
| kid | car | kid | ran | ship | cut | feed |
| top | list | dust | coat | dust | date | car |
| call | car | call | date | pick | pick | ten |
| board | girl | deep | car | test | wide | face |
| gold | year | leave | met | curve | ship | door |
| met | game | sat | feed | dust | talk | face |
| gold | wall | coat | ship | sat | feed | fig |
| coat | game | sat | pool | case | coat | year |
| ball | curve | ball | big | moon | feed | dust |
