# Supplementary material for: Word Frequency Is Associated With Cognitive Effort During Verbal Working Memory: A Functional Near Infrared Spectroscopy (fNIRS) Study
Source: Front Hum Neurosci. 2019 Dec 13;13:433. doi: 10.3389/fnhum.2019.00433 (PMC6923201; doi:10.3389/fnhum.2019.00433)
Supplement: Supplementary file 1 [file Data_Sheet_1.ZIP › Supplementary Material Presentation/Supplementary table legends.docx]

Supplementary Table 1: Low-frequency word lists used for each of the low-frequency word blocks.

Supplementary Table 2: High-frequency word lists used for each of the high-frequency word blocks.
