# Supplementary material for: Word Frequency Is Associated With Cognitive Effort During Verbal Working Memory: A Functional Near Infrared Spectroscopy (fNIRS) Study
Source: Front Hum Neurosci. 2019 Dec 13;13:433. doi: 10.3389/fnhum.2019.00433 (PMC6923201; doi:10.3389/fnhum.2019.00433)
Supplement: Supplementary file 1 [file Data_Sheet_1.ZIP › Supplementary Material Presentation/Supplementary table 1.docx]

| Low List 1 | Low List 2 | Low List 3 | Low List 4 | Low List 5 | Low List 6 | Low List 7 |
| --- | --- | --- | --- | --- | --- | --- |
| badge | badge | badge | pout | knob | rash | bait |
| teal | dab | teal | badge | lab | moat | cove |
| badge | cave | badge | rogue | mitt | rash | bait |
| dank | fin | dank | badge | pawn | moat | wag |
| fang | cave | rib | comb | cove | gag | bait |
| knob | nail | gourd | shack | fin | moat | shin |
| fang | dusk | kilt | comb | bead | beak | bait |
| knob | nail | pup | dusk | cave | rum | gourd |
| fang | curl | rash | perch | moat | beak | wig |
| dusk | gag | pup | dusk | pawn | fizz | fizz |
| mat | mat | mat | gnome | lair | beak | wig |
| dusk | fizz | gourd | cod | rash | gourd | fizz |
| wag | mole | rink | gnome | beak | mitt | peep |
| gourd | nag | gut | mop | dab | gourd | rut |
| wag | lobe | wag | rum | badge | kilt | curl |
| nail | mutt | lair | ledge | chop | mop | lobe |
| lair | foal | wag | kilt | badge | kilt | curl |
| nail | mutt | nail | ledge | chop | perch | mop |
| cove | ledge | lair | beak | pal | teal | weed |
| nail | mug | nail | fizz | chop | perch | nag |
| fang | perch | fade | dank | rum | teal | weed |
| shack | rink | cob | moose | chop | pout | fade |
| fang | perch | fade | gag | dank | wade | cave |
| shack | kilt | shack | moose | sage | pout | poke |
| fang | perch | knob | teal | dank | wade | shack |
| rib | fizz | cart | cuff | mall | shawl | mole |
| gourd | perch | weed | pub | fern | rib | gut |
| mole | fizz | cart | cove | nail | kneel | bead |
| bike | curl | weed | pub | fern | lab | pub |
| rash | rash | rink | fang | knob | mole | goat |
| mat | dusk | mat | pub | fern | lab | shawl |
| cart | gut | cart | chick | knob | sip | goat |
| rink | dank | rink | kite | rogue | cub | rum |
| cart | moat | cart | ledge | shin | shin | comb |
| sage | lobe | shawl | dab | shin | cub | mutt |
| cart | beak | sap | nail | moat | dent | pave |
| ledge | cob | shawl | dab | gill | wade | mutt |
| mitt | foal | fin | foal | moat | dent | pave |
| lobe | din | shawl | pawn | ledge | knob | mutt |
| weed | shag | mop | sap | bead | pub | pave |
| cod | pout | sage | bike | rib | wedge | mutt |
| weed | shag | kite | puck | pawn | cart | sip |
| pad | dusk | pad | bike | shack | lobe | dent |
| dusk | bait | dusk | puck | pawn | wed | chop |
| pad | peep | pad | fern | tease | lobe | dent |
| cart | teal | cart | chick | weed | shack | rib |
| ledge | peep | ledge | fern | gag | beak | kneel |
| sap | wig | cave | chick | rink | nag | rib |
| pave | cove | ledge | goat | cuff | puck | kneel |
| moose | kneel | cave | peep | rink | chop | cuff |
| shawl | cove | fang | goat | dent | puck | kneel |
| ledge | moose | beak | shack | rink | wedge | cart |
| shawl | bead | gut | pout | fizz | gut | pal |
| ledge | fizz | wade | tint | rink | wedge | moose |
| dent | bead | gut | cave | cave | rash | cuff |
| fang | goat | wig | lab | fang | lobe | gnome |
| bead | lab | bead | dusk | gourd | rash | shag |
| chick | fern | chick | lab | pave | moose | wade |
| wig | mole | rash | moat | mug | rash | cart |
| nail | fern | gill | gourd | pave | goat | tint |
| perch | mole | rash | moat | nail | gnome | cart |
| lab | pawn | gill | gourd | pave | pad | teal |
| nag | mat | perch | moat | moose | ledge | knob |
| cave | pawn | chop | gourd | comb | bead | beak |
| gut | gnome | nag | poke | moose | mop | pawn |
| cave | bike | chop | shack | gut | perch | bike |
| gut | weed | sip | weed | moose | mop | pup |
| pad | bike | shack | cod | sip | fern | kilt |
| rash | tint | sip | rum | poke | mop | sage |
| cub | pub | pave | lobe | cub | kilt | lobe |
| rash | cuff | sip | rum | pout | bike | sage |
| wade | pub | fern | sip | gnome | wag | rink |
| teal | rogue | pout | bike | pout | chop | lab |
| shawl | pub | wade | shin | gnome | gill | gourd |
| pup | rogue | pout | teal | goat | fizz | fade |
| rut | shin | shawl | shin | bait | mall | gourd |
| pout | rogue | pout | fade | pal | pave | weed |
| tint | shin | rum | kilt | bait | shin | dent |
| mop | wed | cove | poke | pub | rash | weed |
| tint | fang | tint | lobe | gill | nail | kilt |
| pal | wed | mop | poke | kneel | tint | puck |
| gill | mole | tint | kneel | gill | pawn | rash |
| cuff | fade | pal | poke | mat | tint | moat |
| web | lair | gill | badge | gill | cart | foal |
| dent | rash | web | poke | mitt | tint | pad |
| web | moat | dank | moose | rib | pawn | fin |
| dent | rash | web | tint | mitt | cave | pad |
| mole | nail | mole | moose | sip | pawn | gill |
| fern | shag | fern | poke | cob | badge | lair |
| mole | kite | mole | wag | din | fern | pawn |
| kneel | mole | fern | shawl | fade | mitt | kite |
| din | kite | cove | gourd | din | pub | rum |
| chop | mole | kneel | mall | pout | lobe | sap |
| cuff | sip | din | chop | wig | nail | tease |
| goat | rut | pawn | pawn | shin | foal | wed |
| lab | gourd | goat | tease | pawn | rut | tease |
| goat | wedge | lab | wed | moat | moose | cob |
| curl | gourd | goat | beak | pawn | ledge | rum |
| bun | cove | curl | pave | lab | moose | fern |
